# Supplementary material for: Precision MRI phenotyping enables detection of small changes in body composition for longitudinal cohorts
Source: Sci Rep. 2022 Mar 8;12:3748. doi: 10.1038/s41598-022-07556-y (PMC8904801; doi:10.1038/s41598-022-07556-y)
Supplement: Supplementary file 1 — Supplementary Information. [file 41598_2022_7556_MOESM1_ESM.pdf]

Precision MRI Phenotyping Enables Detection of Small Changes in Body Composition for Longitudinal Cohorts

Brandon Whitcher[1], Marjola Thanaj[1], Madeleine Cule[2], Yi Liu[2], Nicolas Basty[1], Elena P. Sorokin[2], Jimmy D. Bell[1], and E. Louise Thomas[1,*]

[1]Research Centre for Optimal Health, School of Life Sciences, University of Westminster, London, UK
[2]Calico Life Sciences LLC, South San Francisco, California, USA
[*]l.thomas3@westminster.ac.uk

ABSTRACT

Supplementary Information

	Women		Men	
	First Imaging Visit	p-value	First Imaging Visit	p-value
N	23,742		22,167	
N (Cheadle)	13,912		13,399	
N (Newcastle)	5,966		5,311	
Age	63.20 ± 7.52	1.1×10^{-05}*	64.70 ± 7.78	2.4×10^{-10}*
Weight (kg)	69.49 ± 13.48	0.0572	84.12 ± 13.77	0.0132
Height (cm)	162.55 ± 6.25	0.2174	175.86 ± 6.61	8.5×10^{-05}*
BMI (kg/m^2)	26.30 ± 4.89	0.0025	27.18 ± 4.03	6.7×10^{-07}*
Waist Circumference (cm)	83.48 ± 11.98	0.0214	94.90 ± 10.89	9×10^{-05}*
Hip Circumference (cm)	101.54 ± 9.96	0.0349	101.26 ± 7.38	3.2×10^{-06}*
Waist-to-Hip Ratio	0.82 ± 0.07	0.4512	0.94 ± 0.06	0.2387
Systolic BP (mm Hg)	136.57 ± 19.33	0.0865	142.46 ± 17.34	0.1413
Diastolic BP (mm Hg)	77.59 ± 9.91	5.4×10^{-08}*	80.92 ± 9.75	0.0023
Grip Strength (kg)	21.50 ± 5.78	0.0535	36.17 ± 8.58	0.00068

Table S1. All participants with an initial imaging visit for the Cheadle and Newcastle sites. P-values represent group comparisons with the longitudinal cohort from Table 3. An asterisk (*) indicates the p-value is below the significance threshold adjusted for multiple comparisons. BMI: body mass index; BP: blood pressure.

Trait	SNOMED	ICD9	ICD10	Field 20002	Field 20001	Other Fields
Cardiovascular disease (defined by myocardial infraction, stroke and heart failure)	22298006 230690007 84114007	410-412|4289	I20-I25|I60-I64	1074|1075|1076|1081|1086|1491|1583		
Liver disease (defined by chronic viral hepatitis, malignant liver disease, NAFLD and hepatic cirrhosis)	235856003 3738000 197315008 19943007	0700|0702|0703|0704|0709|155|1551|5715|57151|5716|5718|573|5731|5734	B18|C22|K70-K77	1136|1156|1158|1604		
Kidney disease (defined by chronic kidney disease and end-stage renal disease)	709044004 46177005	585|5859	N180-N185|N188|N189	1192|1193		
Type 1 diabetes mellitus	46635009	25001	E10|E100-E109	1222		
Type 2 diabetes mellitus	44054006	25000	E11|E110-E119	1220|1223		
Cancer (all types)	363346000	Type of cancer ICD9 (Field 40013)	Type of cancer ICD10 (Field 40006)		All cancer codes	2453
Metabolic disorder (correspond to carbohydrates and lipids)	75934005	272	E70-E90			
Menopause	289903006	6272	N951	1665		2724

Table S2. Definitions for disease/physiological conditions for cardiovascular disease, liver disease, kidney disease, type-1 and type-2 diabetes mellitus, any cancer, metabolic disorder and menopause. Field 20001: self-reported cancer illness; Field 20002: self-reported non-cancer illness; ICD9: International Classification of Diseases 9th edition; ICD10 International Classification of Diseases 10th edition.

	Dependent variable:						
	Total Muscle	Iliopsoas Muscle	Total Muscle	Total Kidney	Liver	Total Lung	Total Kidney
	(1)	(2)	(3)	(4)	(5)	(6)	(7)
Intercept	16.00***	525.30***	15.98***	261.47***	1,323.56***	2,271.65***	261.70***
	(0.07)	(3.19)	(0.07)	(1.75)	(7.48)	(22.20)	(1.74)
Sex [Male]	5.87***	218.77***	5.89***	56.81***	140.44***	690.36***	56.74***
	(0.10)	(4.63)	(0.10)	(2.60)	(11.17)	(33.16)	(2.60)
Age	−0.10***	−4.39***	−0.10***	−1.38***	−8.98***	5.42***	−1.37***
	(0.005)	(0.22)	(0.005)	(0.12)	(0.49)	(1.46)	(0.12)
BMI	0.53***	15.00***	0.53***	4.32***	38.25***	23.34***	4.33***
	(0.02)	(0.65)	(0.02)	(0.39)	(1.73)	(5.13)	(0.39)
Site	−0.32***	−5.26	−0.31***	−1.02	−9.91	−6.38	−0.99
	(0.07)	(3.17)	(0.07)	(1.67)	(6.80)	(20.19)	(1.67)
Systolic BP	0.002	0.04	0.002	0.06	0.48*	−0.15	0.06
	(0.001)	(0.05)	(0.001)	(0.03)	(0.17)	(0.53)	(0.03)
Diastolic BP	−0.001	−0.07	−0.001	−0.06	0.03	−1.03	−0.06
	(0.002)	(0.08)	(0.002)	(0.06)	(0.31)	(0.94)	(0.06)
ASAT Volume	−0.36***	−10.75***	−0.36***	−0.93*	−19.19***	−62.04***	−0.94*
	(0.02)	(0.66)	(0.02)	(0.41)	(1.84)	(5.50)	(0.41)
VAT Volume	−0.06*	3.19**	−0.06*	0.63	−5.76*	−68.52***	0.69
	(0.02)	(0.96)	(0.02)	(0.58)	(2.62)	(7.52)	(0.58)
Grip Strength	0.02***	0.68***	0.02***				
	(0.002)	(0.08)	(0.002)				
Diagnosis [T2DM]	−0.54**	−22.31*					
	(0.16)	(7.55)					
Diagnosis [CVD]			−0.28*	4.51			
			(0.14)	(3.43)			
Liver Iron					−30.57***		
					(7.99)		
Liver PDFF					17.24***		
					(0.67)		
Diagnosis [Metabolic]					5.38	−76.38	
					(14.90)	(44.41)	
Diagnosis [Liver]							10.20
							(7.71)
Second Imaging Visit	0.04*	0.47	0.03	0.42	15.10***	−25.40*	0.27
	(0.02)	(0.75)	(0.02)	(0.52)	(2.74)	(8.30)	(0.51)
Diagnosis [T2DM] * 2nd Visit	−0.17*	−5.21*					
	(0.07)	(2.59)					
DIagnosis [CVD] * 2nd Visit			−0.05	−3.05			
			(0.05)	(1.65)			
Diagnosis [Metabolic] * 2nd Visit					−23.48*	98.19**	
					(9.38)	(28.59)	
Diagnosis [Liver] * 2nd Visit							−8.39*
							(3.66)
Participants	2,974	2,966	2,974	2,989	2,993	2,994	2,989
Adjusted ICC	0.95	0.96	0.95	0.92	0.84	0.83	0.92
BIC	15,141	50,247	15,159	47,126	61,762	72,446	47,121

Note: $^{*}p<0.05$; $^{**}p<0.001$; $^{***}p<0.00028$

Table S3. Summary of regression coefficients for the linear mixed-effects models in Table 2. Standard errors are provided underneath the regression coefficients. Indicators are appended to the regression coefficients when the p-value is less than 0.05 *, 0.001 ** and 0.00028 *** (Bonferroni corrected). All volumes are in milliliters (ml), execept for total muscle, ASAT and VAT which are in liters (l). ASAT: abdominal subcutaneous adipsose tissue; BIC: Bayesian information criterion; BMI: body mass index; BP: blood pressure; CVD: cardiovascular disease; ICC: intraclass correlation coefficient; PDFF: proton density fat fraction; T2DM: type-2 diabetes mellitus; VAT: visceral adipose tissue.
